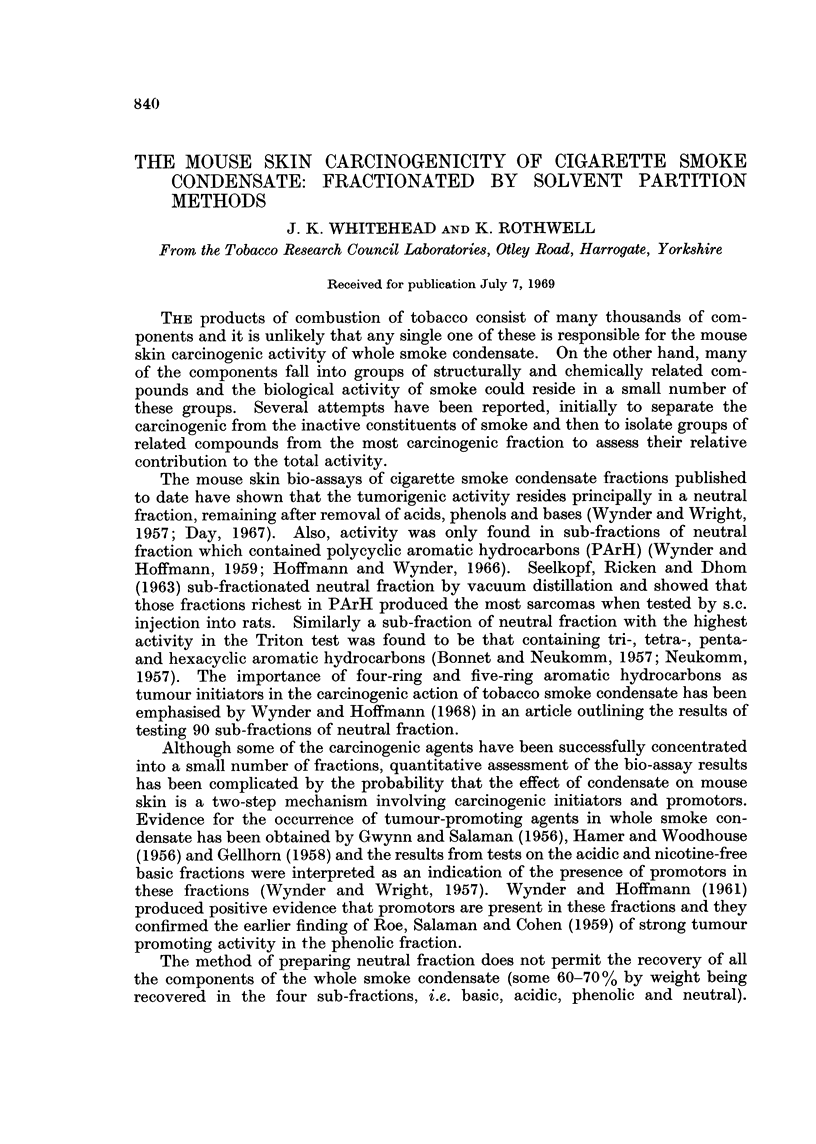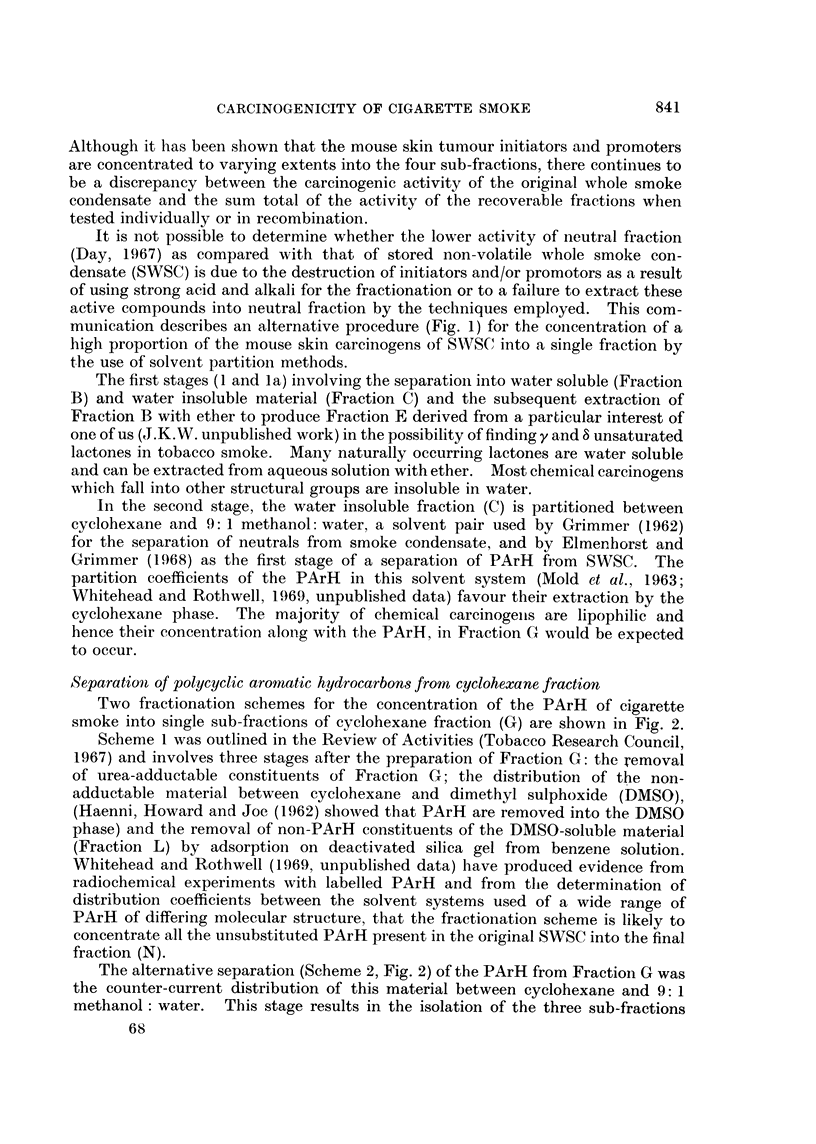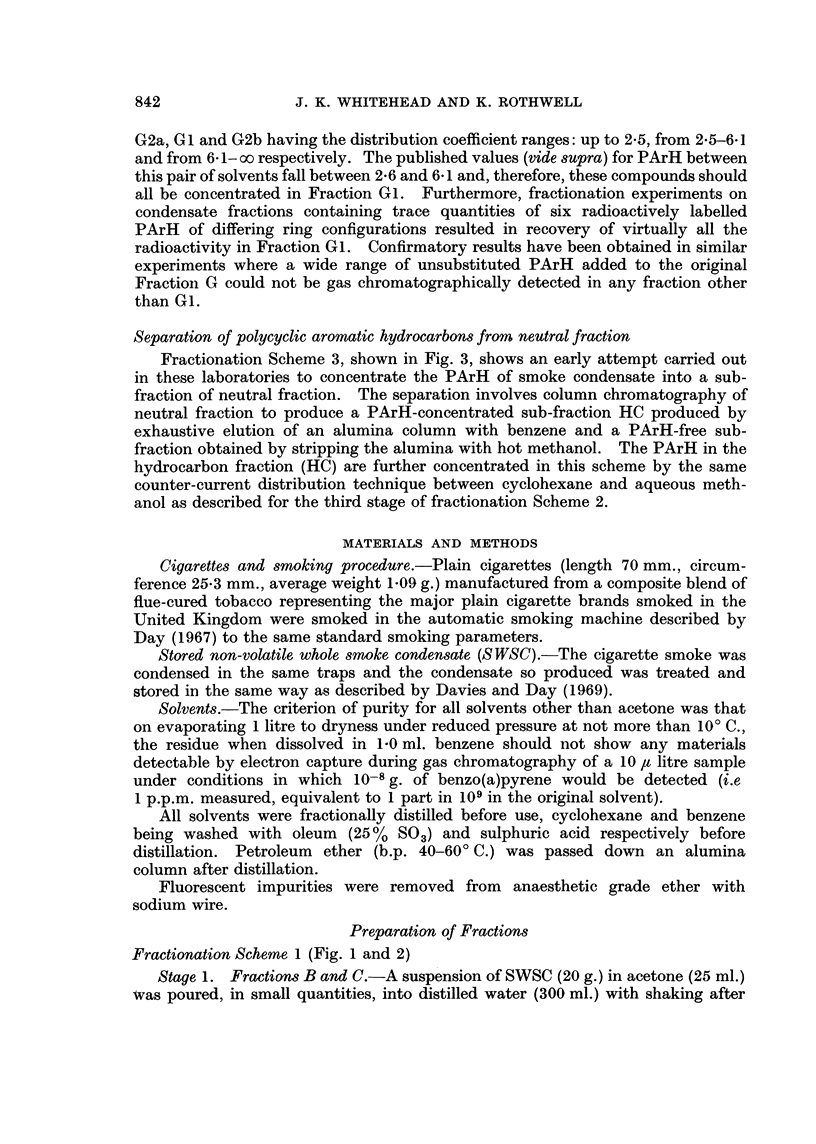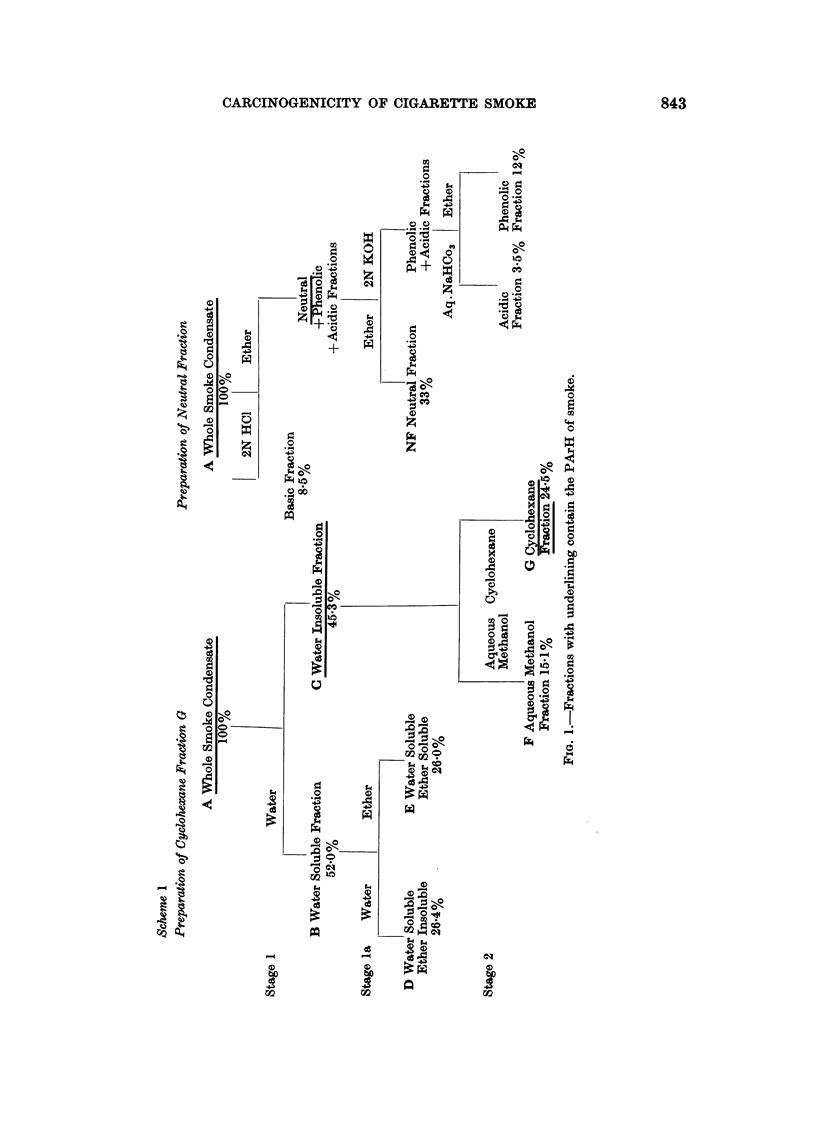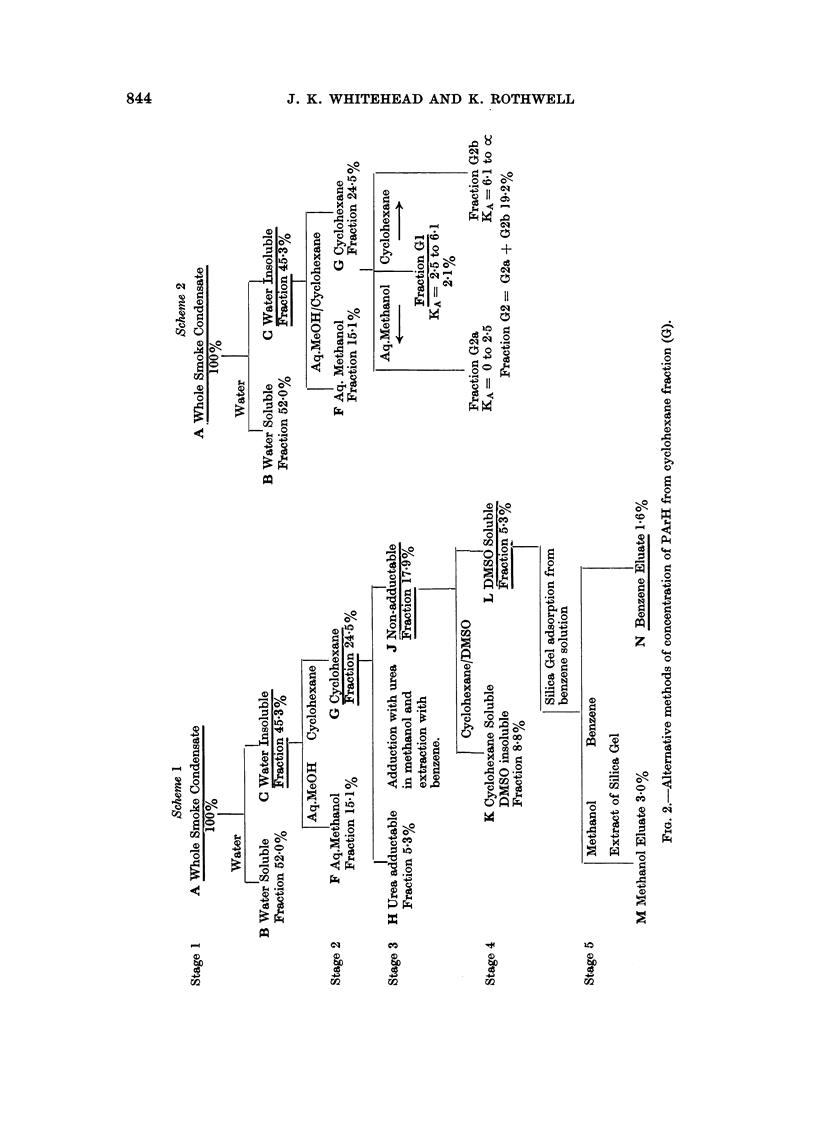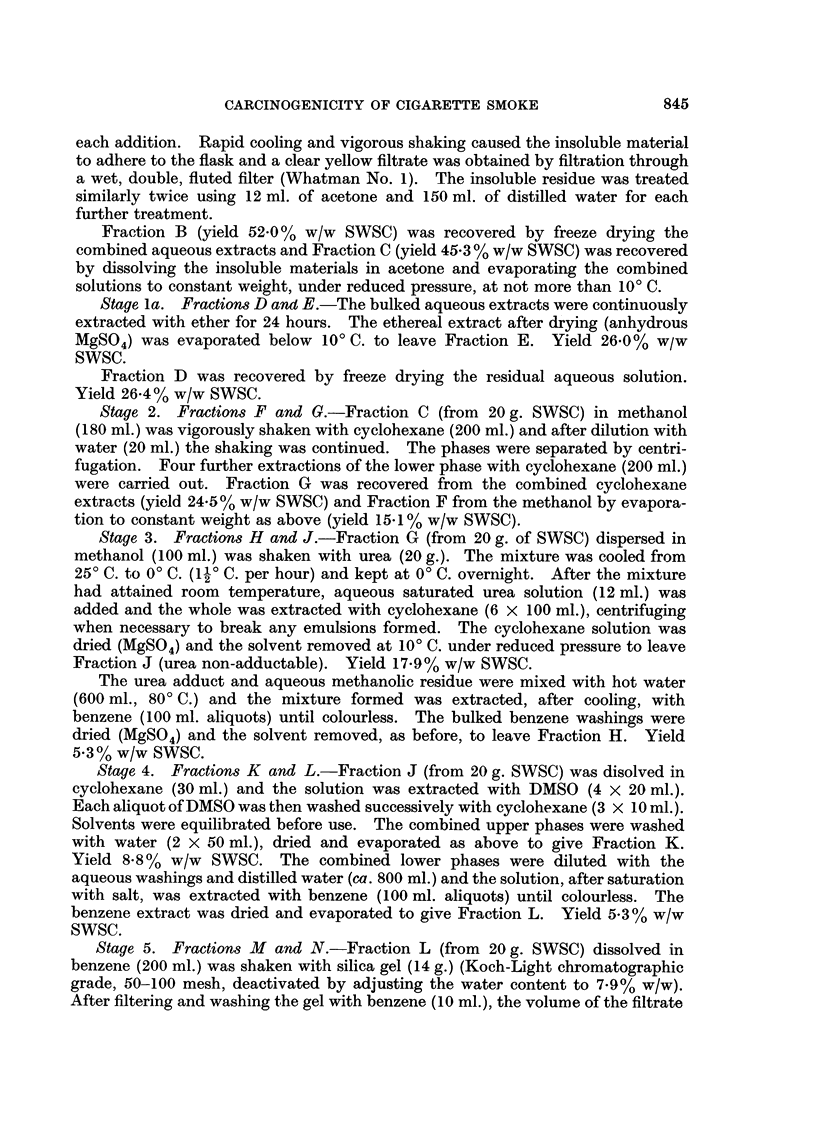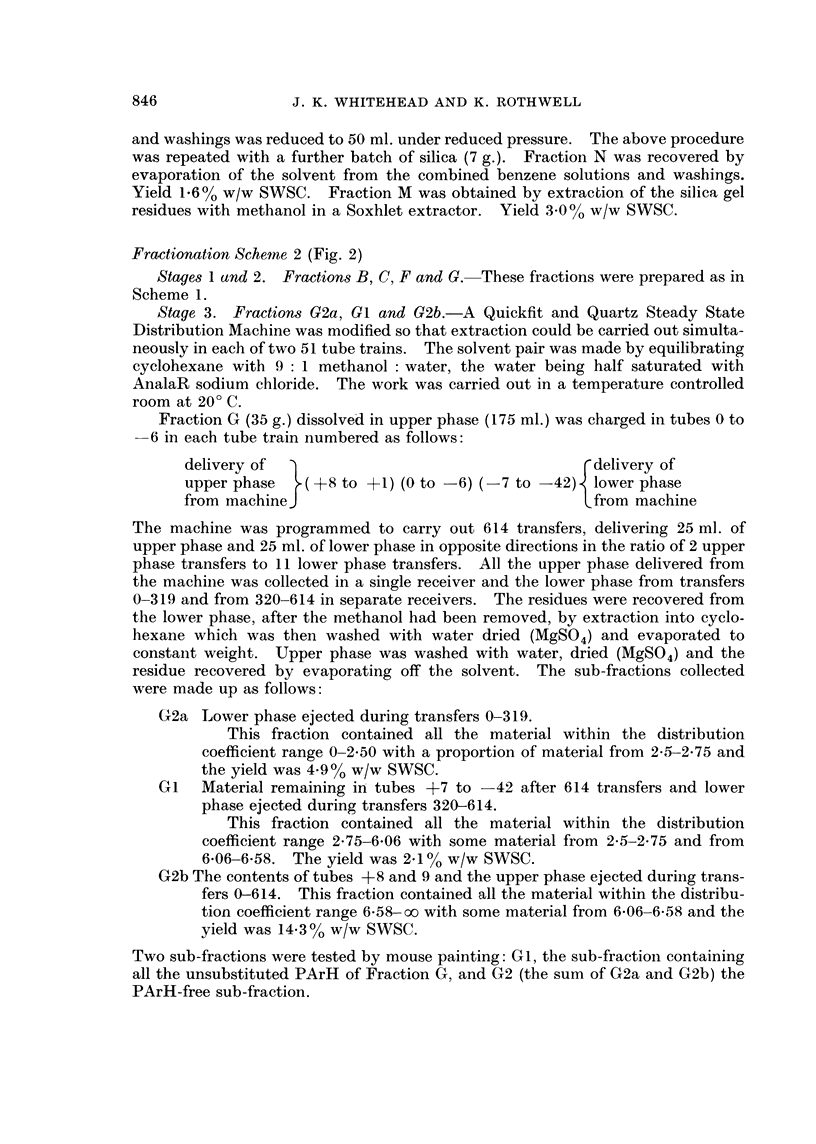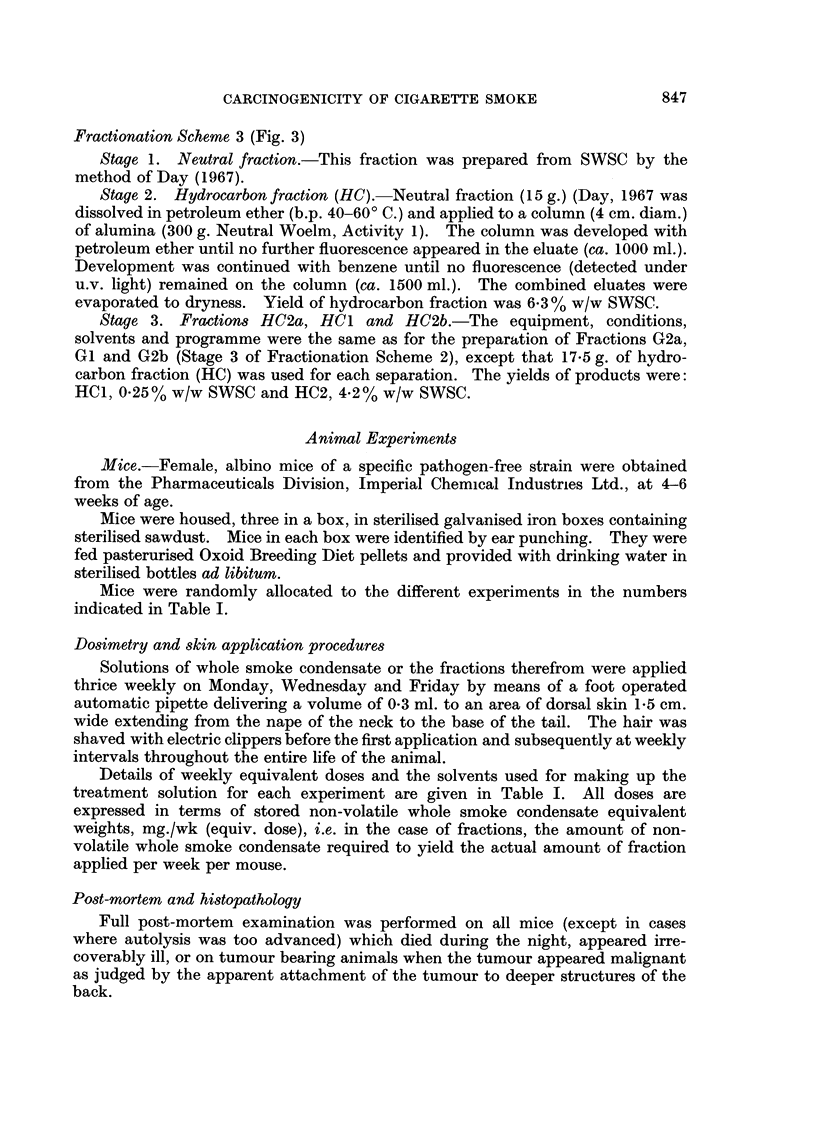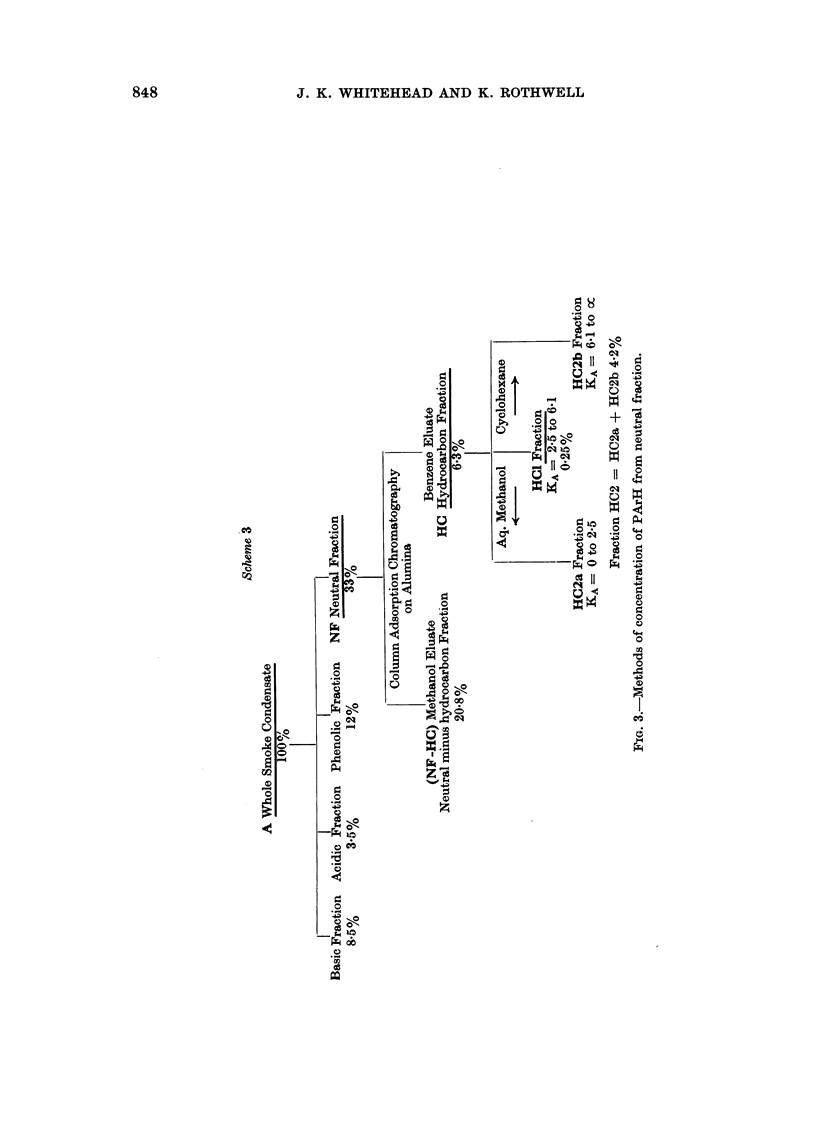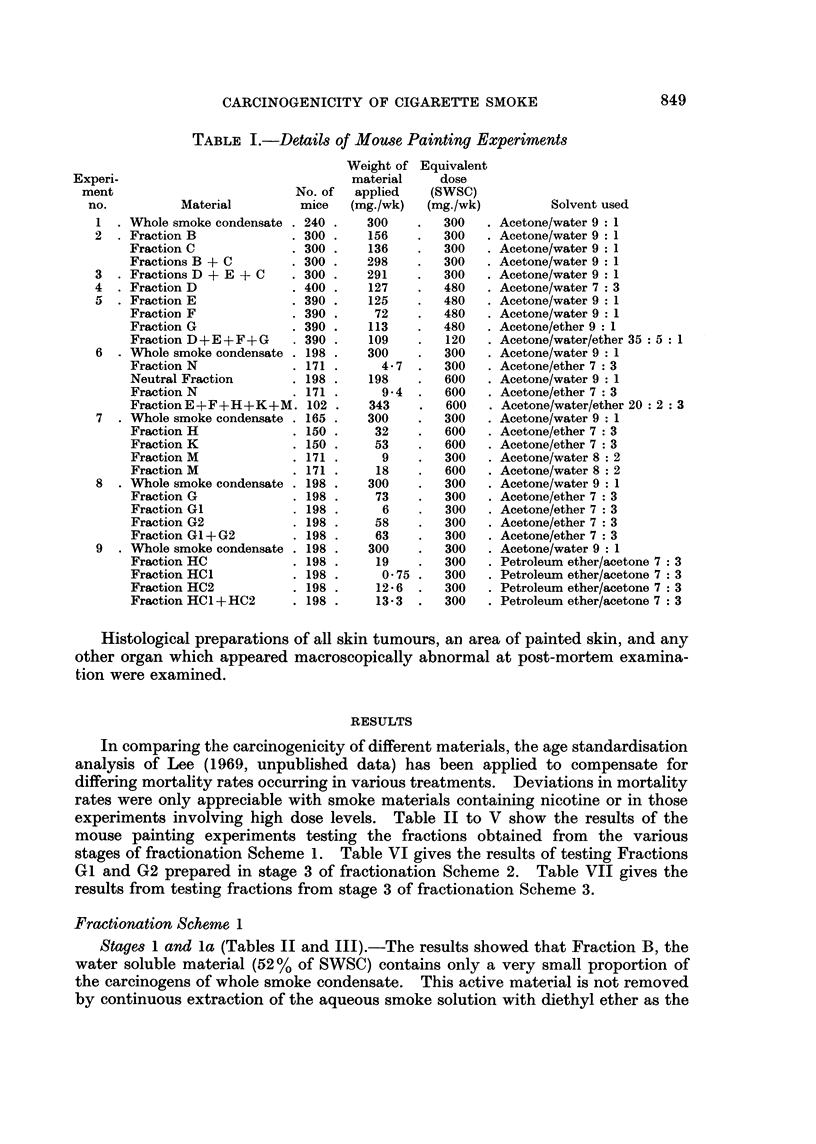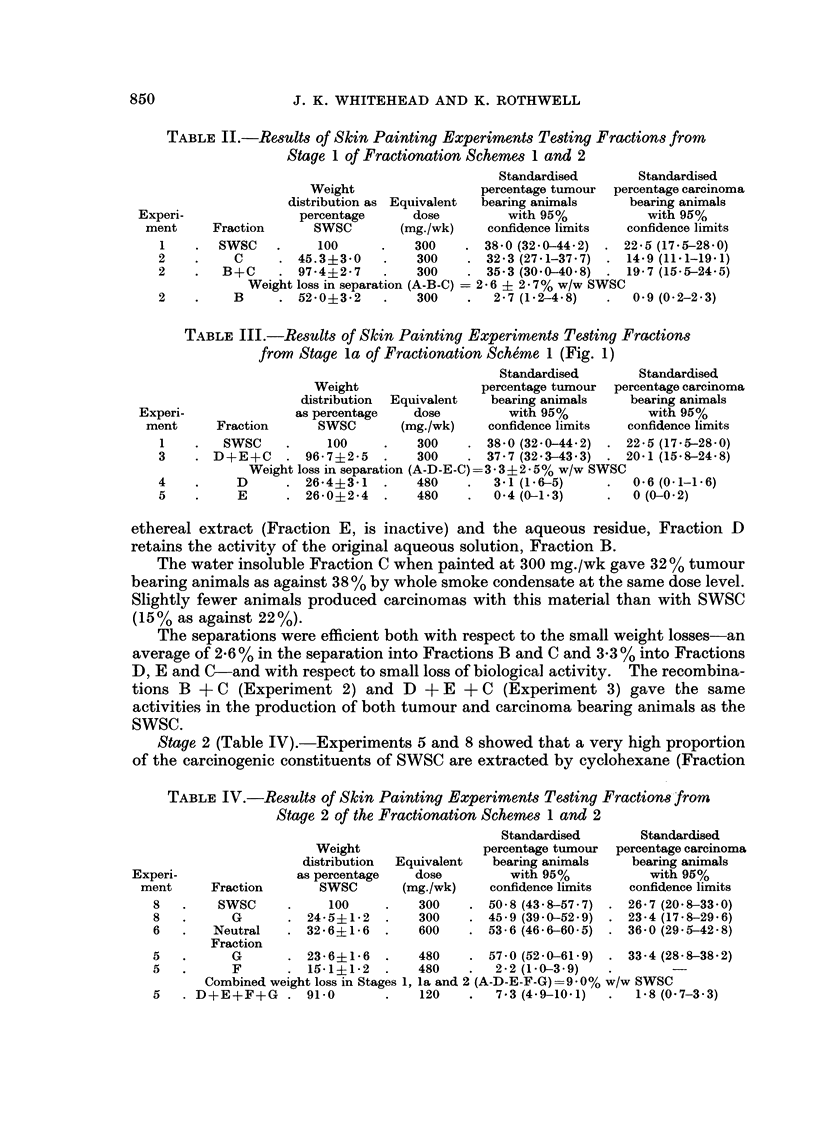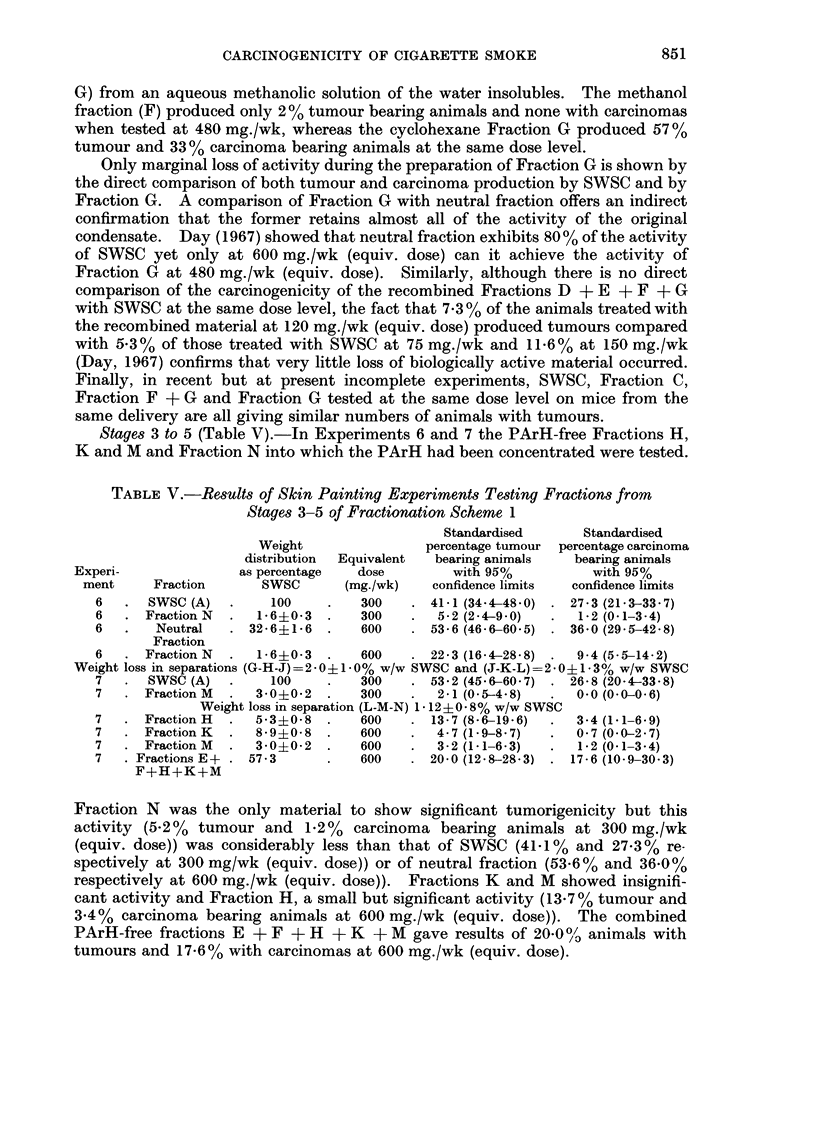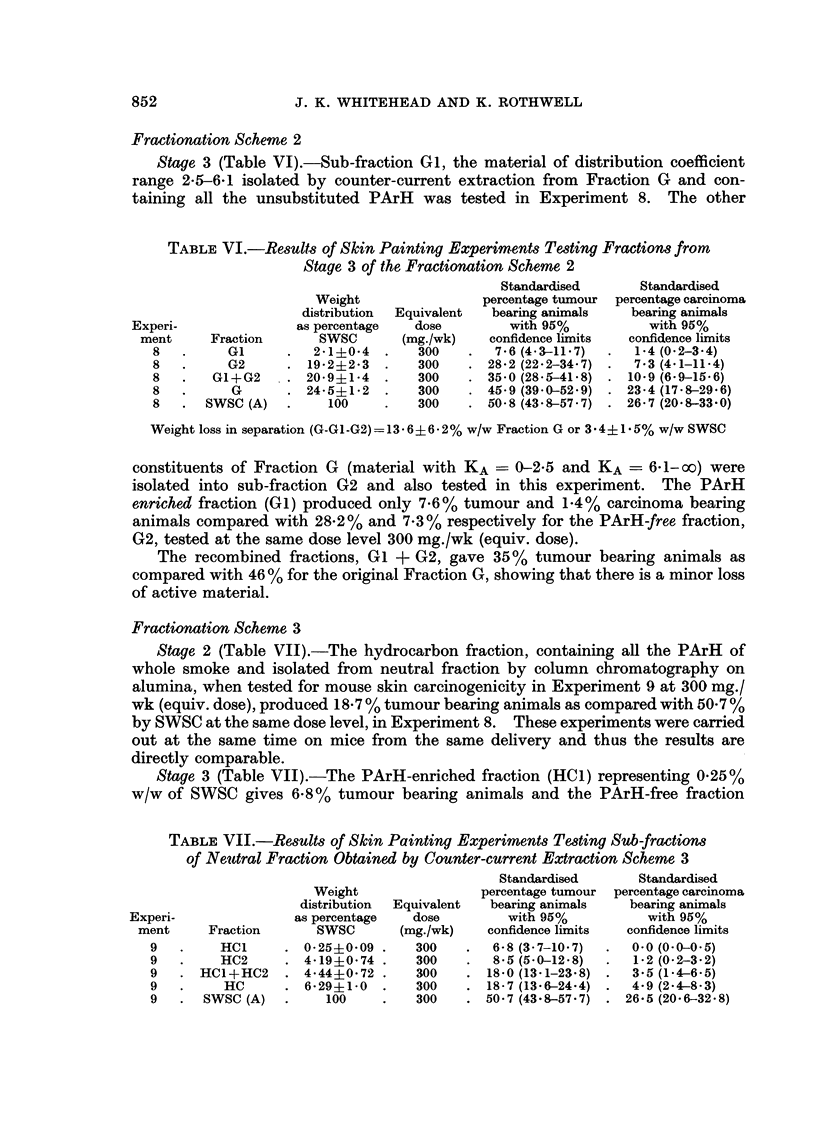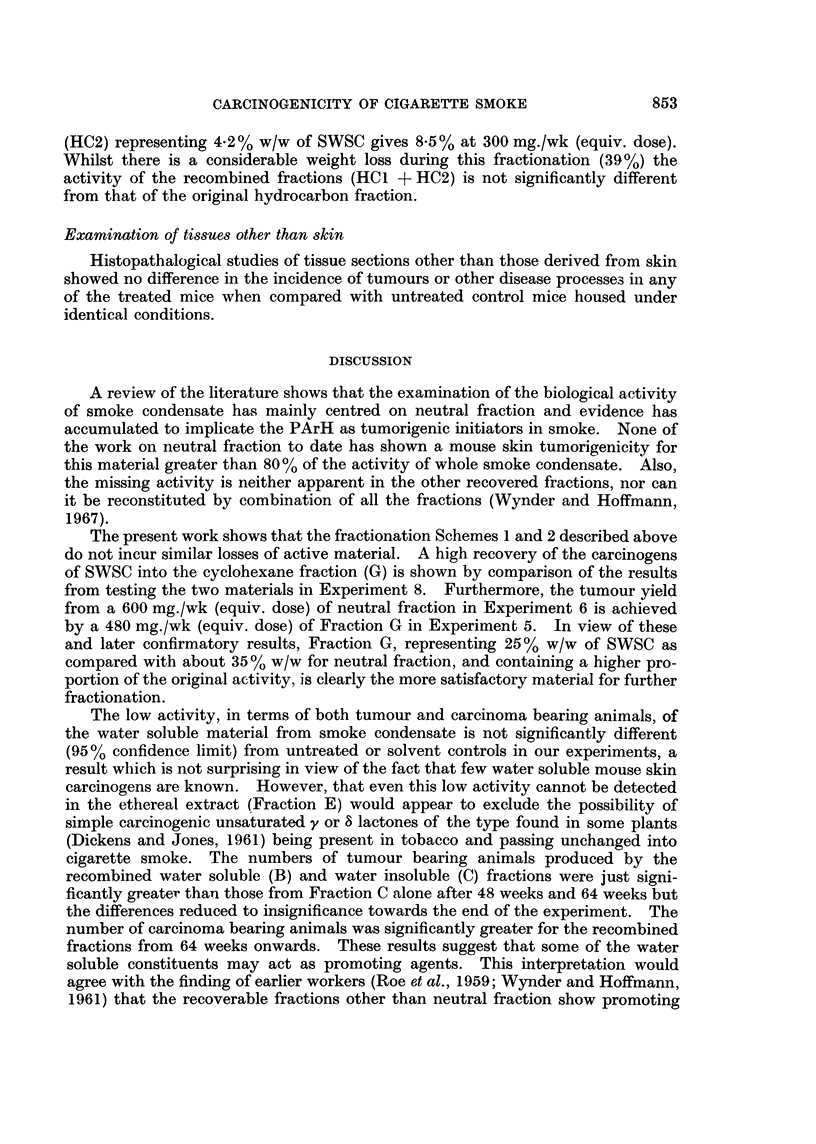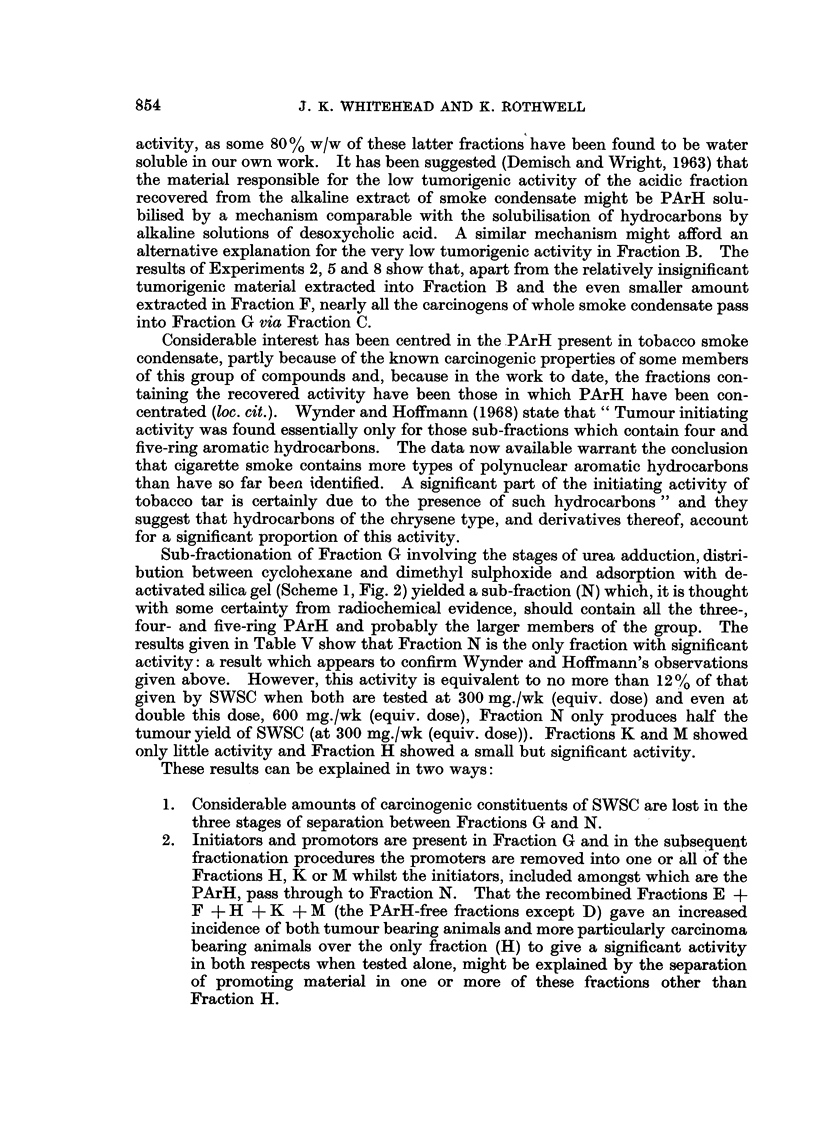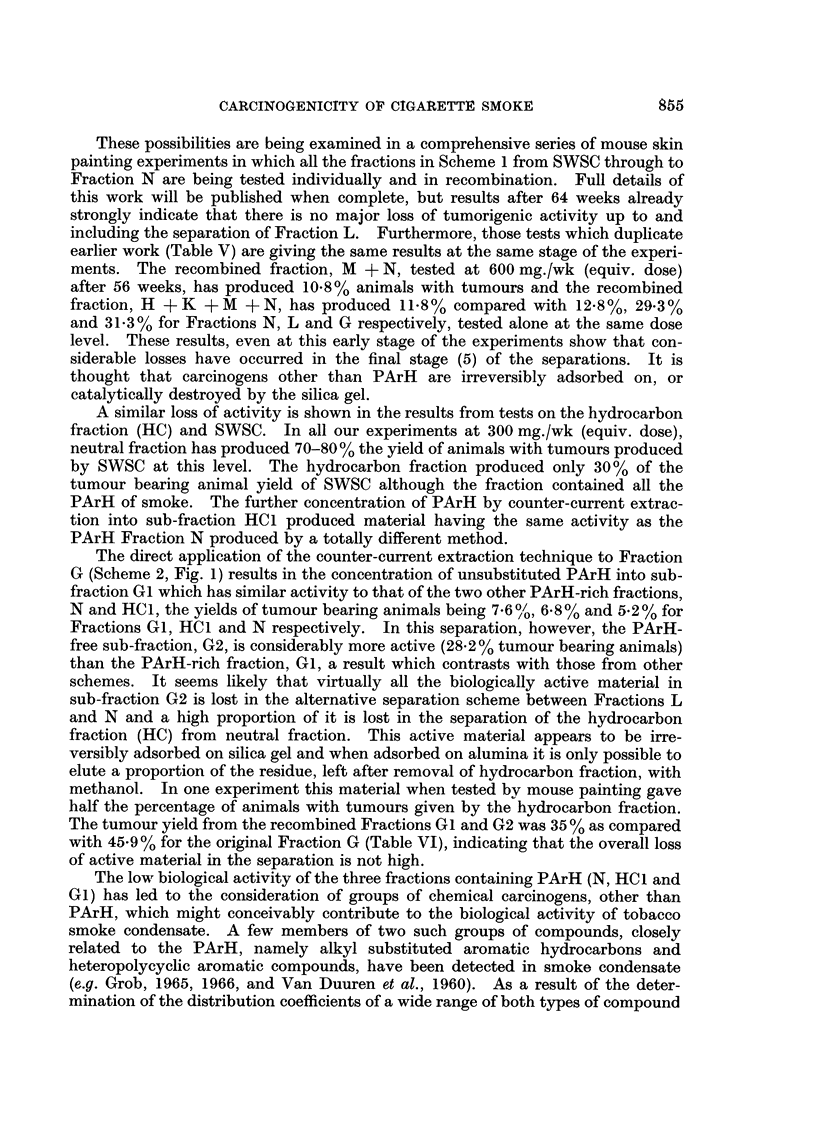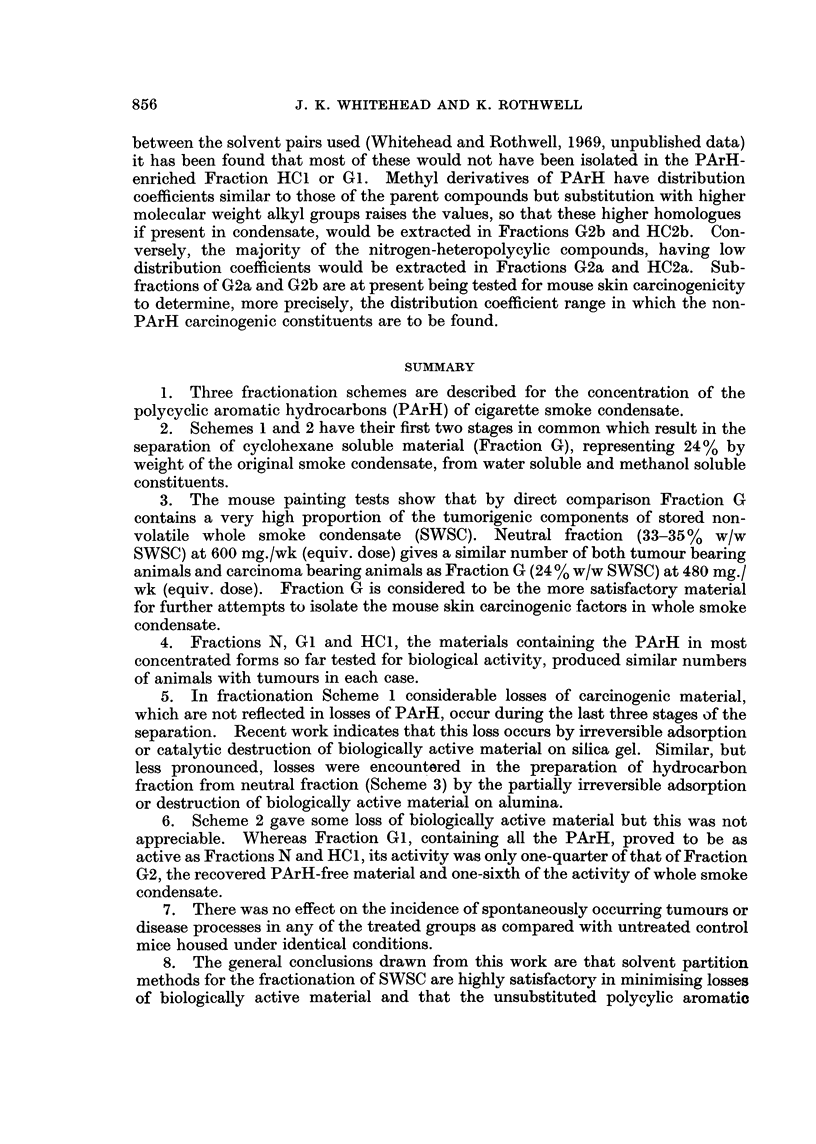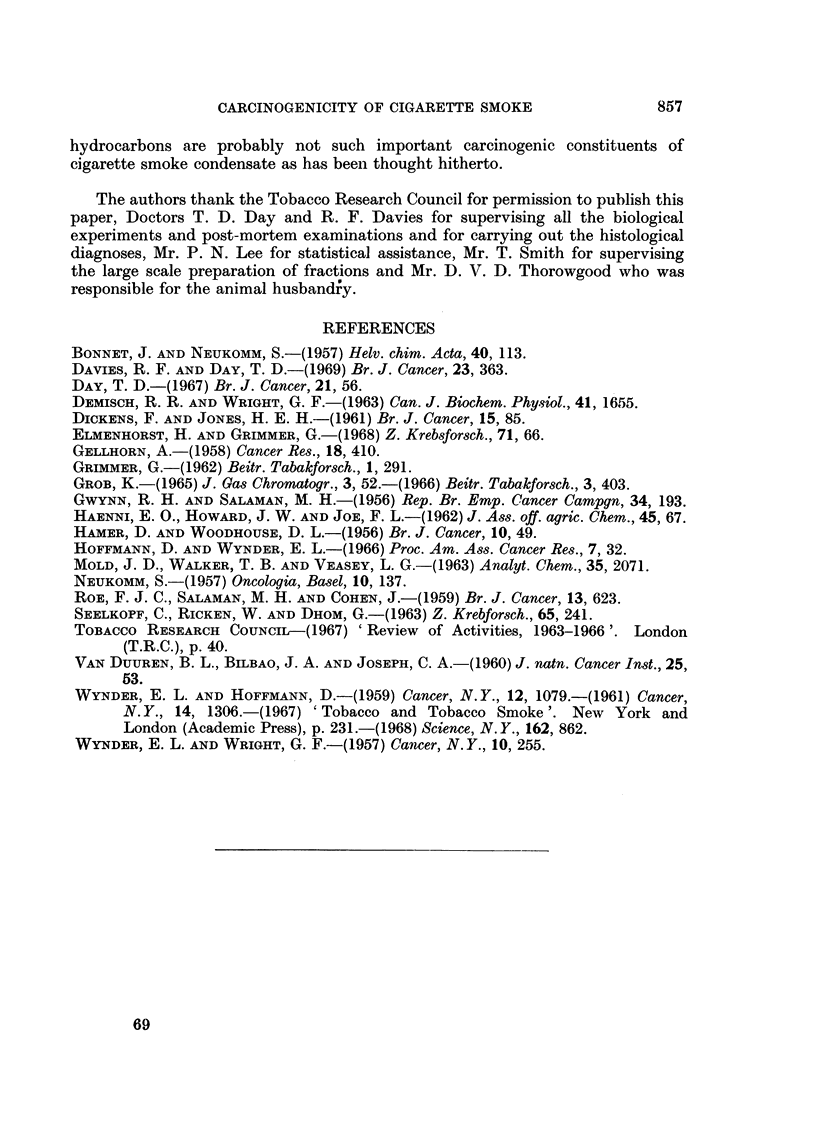# The mouse skin carcinogenicity of cigarette smoke condensate: fractionated by solvent partition methods.

**DOI:** 10.1038/bjc.1969.104

**Published:** 1969-12

**Authors:** J. K. Whitehead, K. Rothwell


### THE MOUSE SKIN CARCINOGENICITY OF CIGARETTE SMOKE CONDENSATE: FRACTIONATED BY SOLVENT PARTITION METHODS

J. K. WHITEHEAD AND K. ROTHWELL

*From the Tobacco Research Council Laboratories, Otley Road, Harrogate, Yorkshire*

Received for publication July 7, 1969

THE products of combustion of tobacco consist of many thousands of components and it is unlikely that any single one of these is responsible for the mouse skin carcinogenic activity of whole smoke condensate. On the other hand, many of the components fall into groups of structurally and chemically related compounds and the biological activity of smoke could reside in a small number of these groups. Several attempts have been reported, initially to separate the carcinogenic from the inactive constituents of smoke and then to isolate groups of related compounds from the most carcinogenic fraction to assess their relative contribution to the total activity.

The mouse skin bio-assays of cigarette smoke condensate fractions published to date have shown that the tumorigenic activity resides principally in a neutral fraction, remaining after removal of acids, phenols and bases (Wynder and Wright, 1957; Day, 1967). Also, activity was only found in sub-fractions of neutral fraction which contained polycyclic aromatic hydrocarbons (PArH) (Wynder and Hoffmann, 1959; Hoffmann and Wynder, 1966). Seelkopf, Ricken and Dhom (1963) sub-fractionated neutral fraction by vacuum distillation and showed that those fractions richest in PArH produced the most sarcomas when tested by s.c. injection into rats. Similarly a sub-fraction of neutral fraction with the highest activity in the Triton test was found to be that containing tri-, tetra-, penta- and hexacyclic aromatic hydrocarbons (Bonnet and Neukomm, 1957; Neukomm, 1957). The importance of four-ring and five-ring aromatic hydrocarbons as tumour initiators in the carcinogenic action of tobacco smoke condensate has been emphasised by Wynder and Hoffmann (1968) in an article outlining the results of testing 90 sub-fractions of neutral fraction.

Although some of the carcinogenic agents have been successfully concentrated into a small number of fractions, quantitative assessment of the bio-assay results has been complicated by the probability that the effect of condensate on mouse skin is a two-step mechanism involving carcinogenic initiators and promotors. Evidence for the occurrence of tumour-promoting agents in whole smoke condensate has been obtained by Gwynn and Salaman (1956), Hamer and Woodhouse (1956) and Gellhorn (1958) and the results from tests on the acidic and nicotine-free basic fractions were interpreted as an indication of the presence of promotors in these fractions (Wynder and Wright, 1957). Wynder and Hoffmann (1961) produced positive evidence that promotors are present in these fractions and they confirmed the earlier finding of Roe, Salaman and Cohen (1959) of strong tumour promoting activity in the phenolic fraction.

The method of preparing neutral fraction does not permit the recovery of all the components of the whole smoke condensate (some 60–70% by weight being recovered in the four sub-fractions, *i.e.* basic, acidic, phenolic and neutral).

Although it has been shown that the mouse skin tumour initiators and promoters are concentrated to varying extents into the four sub-fractions, there continues to be a discrepancy between the carcinogenic activity of the original whole smoke condensate and the sum total of the activity of the recoverable fractions when tested individually or in recombination.

It is not possible to determine whether the lower activity of neutral fraction (Day, 1967) as compared with that of stored non-volatile whole smoke condensate (SWSC) is due to the destruction of initiators and/or promotors as a result of using strong acid and alkali for the fractionation or to a failure to extract these active compounds into neutral fraction by the techniques employed. This communication describes an alternative procedure (Fig. 1) for the concentration of a high proportion of the mouse skin carcinogens of SWSC into a single fraction by the use of solvent partition methods.

The first stages (1 and 1a) involving the separation into water soluble (Fraction B) and water insoluble material (Fraction C) and the subsequent extraction of Fraction B with ether to produce Fraction E derived from a particular interest of one of us (J.K.W. unpublished work) in the possibility of finding $\gamma$ and $\delta$ unsaturated lactones in tobacco smoke. Many naturally occurring lactones are water soluble and can be extracted from aqueous solution with ether. Most chemical carcinogens which fall into other structural groups are insoluble in water.

In the second stage, the water insoluble fraction (C) is partitioned between cyclohexane and 9: 1 methanol: water, a solvent pair used by Grimmer (1962) for the separation of neutrals from smoke condensate, and by Elmenhorst and Grimmer (1968) as the first stage of a separation of PArH from SWSC. The partition coefficients of the PArH in this solvent system (Mold *et al.*, 1963; Whitehead and Rothwell, 1969, unpublished data) favour their extraction by the cyclohexane phase. The majority of chemical carcinogens are lipophilic and hence their concentration along with the PArH, in Fraction G would be expected to occur.

*Separation of polycyclic aromatic hydrocarbons from cyclohexane fraction*

Two fractionation schemes for the concentration of the PArH of cigarette smoke into single sub-fractions of cyclohexane fraction (G) are shown in Fig. 2.

Scheme 1 was outlined in the Review of Activities (Tobacco Research Council, 1967) and involves three stages after the preparation of Fraction G: the removal of urea-adductable constituents of Fraction G; the distribution of the non-adductable material between cyclohexane and dimethyl sulphoxide (DMSO), (Haenni, Howard and Joe (1962) showed that PArH are removed into the DMSO phase) and the removal of non-PArH constituents of the DMSO-soluble material (Fraction L) by adsorption on deactivated silica gel from benzene solution. Whitehead and Rothwell (1969, unpublished data) have produced evidence from radiochemical experiments with labelled PArH and from the determination of distribution coefficients between the solvent systems used of a wide range of PArH of differing molecular structure, that the fractionation scheme is likely to concentrate all the unsubstituted PArH present in the original SWSC into the final fraction (N).

The alternative separation (Scheme 2, Fig. 2) of the PArH from Fraction G was the counter-current distribution of this material between cyclohexane and 9: 1 methanol : water. This stage results in the isolation of the three sub-fractions

G2a, G1 and G2b having the distribution coefficient ranges: up to 2·5, from 2·5–6·1 and from 6·1–∞ respectively. The published values (*vide supra*) for PArH between this pair of solvents fall between 2·6 and 6·1 and, therefore, these compounds should all be concentrated in Fraction G1. Furthermore, fractionation experiments on condensate fractions containing trace quantities of six radioactively labelled PArH of differing ring configurations resulted in recovery of virtually all the radioactivity in Fraction G1. Confirmatory results have been obtained in similar experiments where a wide range of unsubstituted PArH added to the original Fraction G could not be gas chromatographically detected in any fraction other than G1.

*Separation of polycyclic aromatic hydrocarbons from neutral fraction*

Fractionation Scheme 3, shown in Fig. 3, shows an early attempt carried out in these laboratories to concentrate the PArH of smoke condensate into a sub-fraction of neutral fraction. The separation involves column chromatography of neutral fraction to produce a PArH-concentrated sub-fraction HC produced by exhaustive elution of an alumina column with benzene and a PArH-free sub-fraction obtained by stripping the alumina with hot methanol. The PArH in the hydrocarbon fraction (HC) are further concentrated in this scheme by the same counter-current distribution technique between cyclohexane and aqueous methanol as described for the third stage of fractionation Scheme 2.

### MATERIALS AND METHODS

*Cigarettes and smoking procedure.*—Plain cigarettes (length 70 mm., circumference 25·3 mm., average weight 1·09 g.) manufactured from a composite blend of flue-cured tobacco representing the major plain cigarette brands smoked in the United Kingdom were smoked in the automatic smoking machine described by Day (1967) to the same standard smoking parameters.

*Stored non-volatile whole smoke condensate (SWSC).*—The cigarette smoke was condensed in the same traps and the condensate so produced was treated and stored in the same way as described by Davies and Day (1969).

*Solvents.*—The criterion of purity for all solvents other than acetone was that on evaporating 1 litre to dryness under reduced pressure at not more than 10° C., the residue when dissolved in 1·0 ml. benzene should not show any materials detectable by electron capture during gas chromatography of a 10 $\mu$ litre sample under conditions in which $10^{-8}$ g. of benzo(a)pyrene would be detected (*i.e* 1 p.p.m. measured, equivalent to 1 part in $10^9$ in the original solvent).

All solvents were fractionally distilled before use, cyclohexane and benzene being washed with oleum (25% $SO_3$) and sulphuric acid respectively before distillation. Petroleum ether (b.p. 40–60° C.) was passed down an alumina column after distillation.

Fluorescent impurities were removed from anaesthetic grade ether with sodium wire.

### *Preparation of Fractions*

*Fractionation Scheme* 1 (Fig. 1 and 2)

*Stage* 1. *Fractions B and C.*—A suspension of SWSC (20 g.) in acetone (25 ml.) was poured, in small quantities, into distilled water (300 ml.) with shaking after

*Scheme* 1

*Preparation of Cyclohexane Fraction G* — *Preparation of Neutral Fraction*

FIG. 1.—Fractions with underlining contain the PArH of smoke.

FIG. 2.—Alternative methods of concentration of PArH from cyclohexane fraction (G).

each addition. Rapid cooling and vigorous shaking caused the insoluble material to adhere to the flask and a clear yellow filtrate was obtained by filtration through a wet, double, fluted filter (Whatman No. 1). The insoluble residue was treated similarly twice using 12 ml. of acetone and 150 ml. of distilled water for each further treatment.

Fraction B (yield 52·0% w/w SWSC) was recovered by freeze drying the combined aqueous extracts and Fraction C (yield 45·3% w/w SWSC) was recovered by dissolving the insoluble materials in acetone and evaporating the combined solutions to constant weight, under reduced pressure, at not more than 10° C.

*Stage* 1*a*. *Fractions D and E.*—The bulked aqueous extracts were continuously extracted with ether for 24 hours. The ethereal extract after drying (anhydrous $MgSO_4$) was evaporated below 10° C. to leave Fraction E. Yield 26·0% w/w SWSC.

Fraction D was recovered by freeze drying the residual aqueous solution. Yield 26·4% w/w SWSC.

*Stage* 2. *Fractions F and G.*—Fraction C (from 20 g. SWSC) in methanol (180 ml.) was vigorously shaken with cyclohexane (200 ml.) and after dilution with water (20 ml.) the shaking was continued. The phases were separated by centrifugation. Four further extractions of the lower phase with cyclohexane (200 ml.) were carried out. Fraction G was recovered from the combined cyclohexane extracts (yield 24·5% w/w SWSC) and Fraction F from the methanol by evaporation to constant weight as above (yield 15·1% w/w SWSC).

*Stage* 3. *Fractions H and J.*—Fraction G (from 20 g. of SWSC) dispersed in methanol (100 ml.) was shaken with urea (20 g.). The mixture was cooled from 25° C. to 0° C. (1½° C. per hour) and kept at 0° C. overnight. After the mixture had attained room temperature, aqueous saturated urea solution (12 ml.) was added and the whole was extracted with cyclohexane (6 × 100 ml.), centrifuging when necessary to break any emulsions formed. The cyclohexane solution was dried ($MgSO_4$) and the solvent removed at 10° C. under reduced pressure to leave Fraction J (urea non-adductable). Yield 17·9% w/w SWSC.

The urea adduct and aqueous methanolic residue were mixed with hot water (600 ml., 80° C.) and the mixture formed was extracted, after cooling, with benzene (100 ml. aliquots) until colourless. The bulked benzene washings were dried ($MgSO_4$) and the solvent removed, as before, to leave Fraction H. Yield 5·3% w/w SWSC.

*Stage* 4. *Fractions K and L.*—Fraction J (from 20 g. SWSC) was disolved in cyclohexane (30 ml.) and the solution was extracted with DMSO (4 × 20 ml.). Each aliquot of DMSO was then washed successively with cyclohexane (3 × 10 ml.). Solvents were equilibrated before use. The combined upper phases were washed with water (2 × 50 ml.), dried and evaporated as above to give Fraction K. Yield 8·8% w/w SWSC. The combined lower phases were diluted with the aqueous washings and distilled water (*ca*. 800 ml.) and the solution, after saturation with salt, was extracted with benzene (100 ml. aliquots) until colourless. The benzene extract was dried and evaporated to give Fraction L. Yield 5·3% w/w SWSC.

*Stage* 5. *Fractions M and N.*—Fraction L (from 20 g. SWSC) dissolved in benzene (200 ml.) was shaken with silica gel (14 g.) (Koch-Light chromatographic grade, 50–100 mesh, deactivated by adjusting the water content to 7·9% w/w). After filtering and washing the gel with benzene (10 ml.), the volume of the filtrate and washings was reduced to 50 ml. under reduced pressure. The above procedure was repeated with a further batch of silica (7 g.). Fraction N was recovered by evaporation of the solvent from the combined benzene solutions and washings. Yield 1·6% w/w SWSC. Fraction M was obtained by extraction of the silica gel residues with methanol in a Soxhlet extractor. Yield 3·0% w/w SWSC.

*Fractionation Scheme* 2 (Fig. 2)

*Stages* 1 *and* 2. *Fractions B, C, F and G.*—These fractions were prepared as in Scheme 1.

*Stage* 3. *Fractions G2a, G1 and G2b.*—A Quickfit and Quartz Steady State Distribution Machine was modified so that extraction could be carried out simultaneously in each of two 51 tube trains. The solvent pair was made by equilibrating cyclohexane with 9 : 1 methanol : water, the water being half saturated with AnalaR sodium chloride. The work was carried out in a temperature controlled room at 20° C.

Fraction G (35 g.) dissolved in upper phase (175 ml.) was charged in tubes 0 to —6 in each tube train numbered as follows:

delivery of upper phase from machine } ( +8 to +1) (0 to —6) (—7 to —42) { delivery of lower phase from machine

The machine was programmed to carry out 614 transfers, delivering 25 ml. of upper phase and 25 ml. of lower phase in opposite directions in the ratio of 2 upper phase transfers to 11 lower phase transfers. All the upper phase delivered from the machine was collected in a single receiver and the lower phase from transfers 0–319 and from 320–614 in separate receivers. The residues were recovered from the lower phase, after the methanol had been removed, by extraction into cyclohexane which was then washed with water dried ($MgSO_4$) and evaporated to constant weight. Upper phase was washed with water, dried ($MgSO_4$) and the residue recovered by evaporating off the solvent. The sub-fractions collected were made up as follows:

G2a Lower phase ejected during transfers 0–319.
This fraction contained all the material within the distribution coefficient range 0–2·50 with a proportion of material from 2·5–2·75 and the yield was 4·9% w/w SWSC.

G1 Material remaining in tubes +7 to —42 after 614 transfers and lower phase ejected during transfers 320–614.
This fraction contained all the material within the distribution coefficient range 2·75–6·06 with some material from 2·5–2·75 and from 6·06–6·58. The yield was 2·1% w/w SWSC.

G2b The contents of tubes +8 and 9 and the upper phase ejected during transfers 0–614. This fraction contained all the material within the distribution coefficient range 6·58–∞ with some material from 6·06–6·58 and the yield was 14·3% w/w SWSC.

Two sub-fractions were tested by mouse painting: G1, the sub-fraction containing all the unsubstituted PArH of Fraction G, and G2 (the sum of G2a and G2b) the PArH-free sub-fraction.

*Fractionation Scheme* 3 (Fig. 3)

*Stage* 1. *Neutral fraction.*—This fraction was prepared from SWSC by the method of Day (1967).

*Stage* 2. *Hydrocarbon fraction* (*HC*).—Neutral fraction (15 g.) (Day, 1967 was dissolved in petroleum ether (b.p. 40–60° C.) and applied to a column (4 cm. diam.) of alumina (300 g. Neutral Woelm, Activity 1). The column was developed with petroleum ether until no further fluorescence appeared in the eluate (*ca.* 1000 ml.). Development was continued with benzene until no fluorescence (detected under u.v. light) remained on the column (*ca.* 1500 ml.). The combined eluates were evaporated to dryness. Yield of hydrocarbon fraction was 6·3% w/w SWSC.

*Stage* 3. *Fractions HC2a, HC*1 *and HC2b.*—The equipment, conditions, solvents and programme were the same as for the preparation of Fractions G2a, G1 and G2b (Stage 3 of Fractionation Scheme 2), except that 17·5 g. of hydrocarbon fraction (HC) was used for each separation. The yields of products were: HC1, 0·25% w/w SWSC and HC2, 4·2% w/w SWSC.

### *Animal Experiments*

*Mice.*—Female, albino mice of a specific pathogen-free strain were obtained from the Pharmaceuticals Division, Imperial Chemical Industries Ltd., at 4–6 weeks of age.

Mice were housed, three in a box, in sterilised galvanised iron boxes containing sterilised sawdust. Mice in each box were identified by ear punching. They were fed pasterurised Oxoid Breeding Diet pellets and provided with drinking water in sterilised bottles *ad libitum.*

Mice were randomly allocated to the different experiments in the numbers indicated in Table I.

### *Dosimetry and skin application procedures*

Solutions of whole smoke condensate or the fractions therefrom were applied thrice weekly on Monday, Wednesday and Friday by means of a foot operated automatic pipette delivering a volume of 0·3 ml. to an area of dorsal skin 1·5 cm. wide extending from the nape of the neck to the base of the tail. The hair was shaved with electric clippers before the first application and subsequently at weekly intervals throughout the entire life of the animal.

Details of weekly equivalent doses and the solvents used for making up the treatment solution for each experiment are given in Table I. All doses are expressed in terms of stored non-volatile whole smoke condensate equivalent weights, mg./wk (equiv. dose), *i.e.* in the case of fractions, the amount of non-volatile whole smoke condensate required to yield the actual amount of fraction applied per week per mouse.

### *Post-mortem and histopathology*

Full post-mortem examination was performed on all mice (except in cases where autolysis was too advanced) which died during the night, appeared irrecoverably ill, or on tumour bearing animals when the tumour appeared malignant as judged by the apparent attachment of the tumour to deeper structures of the back.

*Scheme* 3

FIG. 3.—Methods of concentration of PArH from neutral fraction.

TABLE I.—*Details of Mouse Painting Experiments*

| Experiment no. | Material | No. of mice | Weight of material applied (mg./wk) | Equivalent dose (SWSC) (mg./wk) | Solvent used |
|---|---|---|---|---|---|
| 1 | Whole smoke condensate | 240 | 300 | 300 | Acetone/water 9 : 1 |
| 2 | Fraction B | 300 | 156 | 300 | Acetone/water 9 : 1 |
| | Fraction C | 300 | 136 | 300 | Acetone/water 9 : 1 |
| | Fractions B + C | 300 | 298 | 300 | Acetone/water 9 : 1 |
| 3 | Fractions D + E + C | 300 | 291 | 300 | Acetone/water 9 : 1 |
| 4 | Fraction D | 400 | 127 | 480 | Acetone/water 7 : 3 |
| 5 | Fraction E | 390 | 125 | 480 | Acetone/water 9 : 1 |
| | Fraction F | 390 | 72 | 480 | Acetone/water 9 : 1 |
| | Fraction G | 390 | 113 | 480 | Acetone/ether 9 : 1 |
| | Fraction D+E+F+G | 390 | 109 | 120 | Acetone/water/ether 35 : 5 : 1 |
| 6 | Whole smoke condensate | 198 | 300 | 300 | Acetone/water 9 : 1 |
| | Fraction N | 171 | 4·7 | 300 | Acetone/ether 7 : 3 |
| | Neutral Fraction | 198 | 198 | 600 | Acetone/water 9 : 1 |
| | Fraction N | 171 | 9·4 | 600 | Acetone/ether 7 : 3 |
| | Fraction E+F+H+K+M | 102 | 343 | 600 | Acetone/water/ether 20 : 2 : 3 |
| 7 | Whole smoke condensate | 165 | 300 | 300 | Acetone/water 9 : 1 |
| | Fraction H | 150 | 32 | 600 | Acetone/ether 7 : 3 |
| | Fraction K | 150 | 53 | 600 | Acetone/ether 7 : 3 |
| | Fraction M | 171 | 9 | 300 | Acetone/water 8 : 2 |
| | Fraction M | 171 | 18 | 600 | Acetone/water 8 : 2 |
| 8 | Whole smoke condensate | 198 | 300 | 300 | Acetone/water 9 : 1 |
| | Fraction G | 198 | 73 | 300 | Acetone/ether 7 : 3 |
| | Fraction G1 | 198 | 6 | 300 | Acetone/ether 7 : 3 |
| | Fraction G2 | 198 | 58 | 300 | Acetone/ether 7 : 3 |
| | Fraction G1+G2 | 198 | 63 | 300 | Acetone/ether 7 : 3 |
| 9 | Whole smoke condensate | 198 | 300 | 300 | Acetone/water 9 : 1 |
| | Fraction HC | 198 | 19 | 300 | Petroleum ether/acetone 7 : 3 |
| | Fraction HC1 | 198 | 0·75 | 300 | Petroleum ether/acetone 7 : 3 |
| | Fraction HC2 | 198 | 12·6 | 300 | Petroleum ether/acetone 7 : 3 |
| | Fraction HC1+HC2 | 198 | 13·3 | 300 | Petroleum ether/acetone 7 : 3 |

Histological preparations of all skin tumours, an area of painted skin, and any other organ which appeared macroscopically abnormal at post-mortem examination were examined.

### RESULTS

In comparing the carcinogenicity of different materials, the age standardisation analysis of Lee (1969, unpublished data) has been applied to compensate for differing mortality rates occurring in various treatments. Deviations in mortality rates were only appreciable with smoke materials containing nicotine or in those experiments involving high dose levels. Table II to V show the results of the mouse painting experiments testing the fractions obtained from the various stages of fractionation Scheme 1. Table VI gives the results of testing Fractions G1 and G2 prepared in stage 3 of fractionation Scheme 2. Table VII gives the results from testing fractions from stage 3 of fractionation Scheme 3.

### *Fractionation Scheme* 1

*Stages* 1 *and* 1*a* (Tables II and III).—The results showed that Fraction B, the water soluble material (52% of SWSC) contains only a very small proportion of the carcinogens of whole smoke condensate. This active material is not removed by continuous extraction of the aqueous smoke solution with diethyl ether as the

TABLE II.—*Results of Skin Painting Experiments Testing Fractions from Stage* 1 *of Fractionation Schemes* 1 *and* 2

| Experiment | Fraction | Weight distribution as percentage SWSC | Equivalent dose (mg./wk) | Standardised percentage tumour bearing animals with 95% confidence limits | Standardised percentage carcinoma bearing animals with 95% confidence limits |
|---|---|---|---|---|---|
| 1 | SWSC | 100 | 300 | 38·0 (32·0–44·2) | 22·5 (17·5–28·0) |
| 2 | C | 45.3±3·0 | 300 | 32·3 (27·1–37·7) | 14·9 (11·1–19·1) |
| 2 | B+C | 97·4±2·7 | 300 | 35·3 (30·0–40·8) | 19·7 (15·5–24·5) |
| | Weight loss in separation (A-B-C) = 2·6 ± 2·7% w/w SWSC | | | | |
| 2 | B | 52·0±3·2 | 300 | 2·7 (1·2–4·8) | 0·9 (0·2–2·3) |

TABLE III.—*Results of Skin Painting Experiments Testing Fractions from Stage* 1a *of Fractionation Schéme* 1 (Fig. 1)

| Experiment | Fraction | Weight distribution as percentage SWSC | Equivalent dose (mg./wk) | Standardised percentage tumour bearing animals with 95% confidence limits | Standardised percentage carcinoma bearing animals with 95% confidence limits |
|---|---|---|---|---|---|
| 1 | SWSC | 100 | 300 | 38·0 (32·0–44·2) | 22·5 (17·5–28·0) |
| 3 | D+E+C | 96·7±2·5 | 300 | 37·7 (32·3–43·3) | 20·1 (15·8–24·8) |
| | Weight loss in separation (A-D-E-C)=3·3±2·5% w/w SWSC | | | | |
| 4 | D | 26·4±3·1 | 480 | 3·1 (1·6–5) | 0·6 (0·1–1·6) |
| 5 | E | 26·0±2·4 | 480 | 0·4 (0–1·3) | 0 (0–0·2) |

ethereal extract (Fraction E, is inactive) and the aqueous residue, Fraction D retains the activity of the original aqueous solution, Fraction B.

The water insoluble Fraction C when painted at 300 mg./wk gave 32% tumour bearing animals as against 38% by whole smoke condensate at the same dose level. Slightly fewer animals produced carcinomas with this material than with SWSC (15% as against 22%).

The separations were efficient both with respect to the small weight losses—an average of 2·6% in the separation into Fractions B and C and 3·3% into Fractions D, E and C—and with respect to small loss of biological activity. The recombinations B + C (Experiment 2) and D + E + C (Experiment 3) gave the same activities in the production of both tumour and carcinoma bearing animals as the SWSC.

*Stage* 2 (Table IV).—Experiments 5 and 8 showed that a very high proportion of the carcinogenic constituents of SWSC are extracted by cyclohexane (Fraction

TABLE IV.—*Results of Skin Painting Experiments Testing Fractions from Stage* 2 *of the Fractionation Schemes* 1 *and* 2

| Experiment | Fraction | Weight distribution as percentage SWSC | Equivalent dose (mg./wk) | Standardised percentage tumour bearing animals with 95% confidence limits | Standardised percentage carcinoma bearing animals with 95% confidence limits |
|---|---|---|---|---|---|
| 8 | SWSC | 100 | 300 | 50·8 (43·8–57·7) | 26·7 (20·8–33·0) |
| 8 | G | 24·5±1·2 | 300 | 45·9 (39·0–52·9) | 23·4 (17·8–29·6) |
| 6 | Neutral Fraction | 32·6±1·6 | 600 | 53·6 (46·6–60·5) | 36·0 (29·5–42·8) |
| 5 | G | 23·6±1·6 | 480 | 57·0 (52·0–61·9) | 33·4 (28·8–38·2) |
| 5 | F | 15·1±1·2 | 480 | 2·2 (1·0–3·9) | — |
| | Combined weight loss in Stages 1, 1a and 2 (A-D-E-F-G)=9·0% w/w SWSC | | | | |
| 5 | D+E+F+G | 91·0 | 120 | 7·3 (4·9–10·1) | 1·8 (0·7–3·3) |

G) from an aqueous methanolic solution of the water insolubles. The methanol fraction (F) produced only 2% tumour bearing animals and none with carcinomas when tested at 480 mg./wk, whereas the cyclohexane Fraction G produced 57% tumour and 33% carcinoma bearing animals at the same dose level.

Only marginal loss of activity during the preparation of Fraction G is shown by the direct comparison of both tumour and carcinoma production by SWSC and by Fraction G. A comparison of Fraction G with neutral fraction offers an indirect confirmation that the former retains almost all of the activity of the original condensate. Day (1967) showed that neutral fraction exhibits 80% of the activity of SWSC yet only at 600 mg./wk (equiv. dose) can it achieve the activity of Fraction G at 480 mg./wk (equiv. dose). Similarly, although there is no direct comparison of the carcinogenicity of the recombined Fractions D + E + F + G with SWSC at the same dose level, the fact that 7·3% of the animals treated with the recombined material at 120 mg./wk (equiv. dose) produced tumours compared with 5·3% of those treated with SWSC at 75 mg./wk and 11·6% at 150 mg./wk (Day, 1967) confirms that very little loss of biologically active material occurred. Finally, in recent but at present incomplete experiments, SWSC, Fraction C, Fraction F + G and Fraction G tested at the same dose level on mice from the same delivery are all giving similar numbers of animals with tumours.

*Stages* 3 *to* 5 (Table V).—In Experiments 6 and 7 the PArH-free Fractions H, K and M and Fraction N into which the PArH had been concentrated were tested.

TABLE V.—*Results of Skin Painting Experiments Testing Fractions from Stages* 3–5 *of Fractionation Scheme* 1

| Experiment | Fraction | Weight distribution as percentage SWSC | Equivalent dose (mg./wk) | Standardised percentage tumour bearing animals with 95% confidence limits | Standardised percentage carcinoma bearing animals with 95% confidence limits |
|---|---|---|---|---|---|
| 6 | SWSC (A) | 100 | 300 | 41·1 (34·4–48·0) | 27·3 (21·3–33·7) |
| 6 | Fraction N | 1·6±0·3 | 300 | 5·2 (2·4–9·0) | 1·2 (0·1–3·4) |
| 6 | Neutral Fraction | 32·6±1·6 | 600 | 53·6 (46·6–60·5) | 36·0 (29·5–42·8) |
| 6 | Fraction N | 1·6±0·3 | 600 | 22·3 (16·4–28·8) | 9·4 (5·5–14·2) |
| Weight loss in separations (G-H-J)=2·0±1·0% w/w SWSC and (J-K-L)=2·0±1·3% w/w SWSC | | | | | |
| 7 | SWSC (A) | 100 | 300 | 53·2 (45·6–60·7) | 26·8 (20·4–33·8) |
| 7 | Fraction M | 3·0±0·2 | 300 | 2·1 (0·5–4·8) | 0·0 (0·0–0·6) |
| Weight loss in separation (L-M-N) 1·12±0·8% w/w SWSC | | | | | |
| 7 | Fraction H | 5·3±0·8 | 600 | 13·7 (8·6–19·6) | 3·4 (1·1–6·9) |
| 7 | Fraction K | 8·9±0·8 | 600 | 4·7 (1·9–8·7) | 0·7 (0·0–2·7) |
| 7 | Fraction M | 3·0±0·2 | 600 | 3·2 (1·1–6·3) | 1·2 (0·1–3·4) |
| 7 | Fractions E+F+H+K+M | 57·3 | 600 | 20·0 (12·8–28·3) | 17·6 (10·9–30·3) |

Fraction N was the only material to show significant tumorigenicity but this activity (5·2% tumour and 1·2% carcinoma bearing animals at 300 mg./wk (equiv. dose)) was considerably less than that of SWSC (41·1% and 27·3% respectively at 300 mg/wk (equiv. dose)) or of neutral fraction (53·6% and 36·0% respectively at 600 mg./wk (equiv. dose)). Fractions K and M showed insignificant activity and Fraction H, a small but significant activity (13·7% tumour and 3·4% carcinoma bearing animals at 600 mg./wk (equiv. dose)). The combined PArH-free fractions E + F + H + K + M gave results of 20·0% animals with tumours and 17·6% with carcinomas at 600 mg./wk (equiv. dose).

*Fractionation Scheme* 2

*Stage* 3 (Table VI).—Sub-fraction G1, the material of distribution coefficient range 2·5–6·1 isolated by counter-current extraction from Fraction G and containing all the unsubstituted PArH was tested in Experiment 8. The other

TABLE VI.—*Results of Skin Painting Experiments Testing Fractions from Stage* 3 *of the Fractionation Scheme* 2

| Experiment | Fraction | Weight distribution as percentage SWSC | Equivalent dose (mg./wk) | Standardised percentage tumour bearing animals with 95% confidence limits | Standardised percentage carcinoma bearing animals with 95% confidence limits |
|---|---|---|---|---|---|
| 8 | G1 | 2·1±0·4 | 300 | 7·6 (4·3–11·7) | 1·4 (0·2–3·4) |
| 8 | G2 | 19·2±2·3 | 300 | 28·2 (22·2–34·7) | 7·3 (4·1–11·4) |
| 8 | G1+G2 | 20·9±1·4 | 300 | 35·0 (28·5–41·8) | 10·9 (6·9–15·6) |
| 8 | G | 24·5±1·2 | 300 | 45·9 (39·0–52·9) | 23·4 (17·8–29·6) |
| 8 | SWSC (A) | 100 | 300 | 50·8 (43·8–57·7) | 26·7 (20·8–33·0) |

Weight loss in separation (G-G1-G2)=13·6±6·2% w/w Fraction G or 3·4±1·5% w/w SWSC

constituents of Fraction G (material with $K_A$ = 0–2·5 and $K_A$ = 6·1–∞) were isolated into sub-fraction G2 and also tested in this experiment. The PArH *enriched* fraction (G1) produced only 7·6% tumour and 1·4% carcinoma bearing animals compared with 28·2% and 7·3% respectively for the PArH-*free* fraction, G2, tested at the same dose level 300 mg./wk (equiv. dose).

The recombined fractions, G1 + G2, gave 35% tumour bearing animals as compared with 46% for the original Fraction G, showing that there is a minor loss of active material.

*Fractionation Scheme* 3

*Stage* 2 (Table VII).—The hydrocarbon fraction, containing all the PArH of whole smoke and isolated from neutral fraction by column chromatography on alumina, when tested for mouse skin carcinogenicity in Experiment 9 at 300 mg./wk (equiv. dose), produced 18·7% tumour bearing animals as compared with 50·7% by SWSC at the same dose level, in Experiment 8. These experiments were carried out at the same time on mice from the same delivery and thus the results are directly comparable.

*Stage* 3 (Table VII).—The PArH-enriched fraction (HC1) representing 0·25% w/w of SWSC gives 6·8% tumour bearing animals and the PArH-free fraction

TABLE VII.—*Results of Skin Painting Experiments Testing Sub-fractions of Neutral Fraction Obtained by Counter-current Extraction Scheme* 3

| Experiment | Fraction | Weight distribution as percentage SWSC | Equivalent dose (mg./wk) | Standardised percentage tumour bearing animals with 95% confidence limits | Standardised percentage carcinoma bearing animals with 95% confidence limits |
|---|---|---|---|---|---|
| 9 | HC1 | 0·25±0·09 | 300 | 6·8 (3·7–10·7) | 0·0 (0·0–0·5) |
| 9 | HC2 | 4·19±0·74 | 300 | 8·5 (5·0–12·8) | 1·2 (0·2–3·2) |
| 9 | HC1+HC2 | 4·44±0·72 | 300 | 18·0 (13·1–23·8) | 3·5 (1·4–6·5) |
| 9 | HC | 6·29±1·0 | 300 | 18·7 (13·6–24·4) | 4·9 (2·4–8·3) |
| 9 | SWSC (A) | 100 | 300 | 50·7 (43·8–57·7) | 26·5 (20·6–32·8) |

(HC2) representing 4·2% w/w of SWSC gives 8·5% at 300 mg./wk (equiv. dose). Whilst there is a considerable weight loss during this fractionation (39%) the activity of the recombined fractions (HC1 + HC2) is not significantly different from that of the original hydrocarbon fraction.

*Examination of tissues other than skin*

Histopathalogical studies of tissue sections other than those derived from skin showed no difference in the incidence of tumours or other disease processes in any of the treated mice when compared with untreated control mice housed under identical conditions.

### DISCUSSION

A review of the literature shows that the examination of the biological activity of smoke condensate has mainly centred on neutral fraction and evidence has accumulated to implicate the PArH as tumorigenic initiators in smoke. None of the work on neutral fraction to date has shown a mouse skin tumorigenicity for this material greater than 80% of the activity of whole smoke condensate. Also, the missing activity is neither apparent in the other recovered fractions, nor can it be reconstituted by combination of all the fractions (Wynder and Hoffmann, 1967).

The present work shows that the fractionation Schemes 1 and 2 described above do not incur similar losses of active material. A high recovery of the carcinogens of SWSC into the cyclohexane fraction (G) is shown by comparison of the results from testing the two materials in Experiment 8. Furthermore, the tumour yield from a 600 mg./wk (equiv. dose) of neutral fraction in Experiment 6 is achieved by a 480 mg./wk (equiv. dose) of Fraction G in Experiment 5. In view of these and later confirmatory results, Fraction G, representing 25% w/w of SWSC as compared with about 35% w/w for neutral fraction, and containing a higher proportion of the original activity, is clearly the more satisfactory material for further fractionation.

The low activity, in terms of both tumour and carcinoma bearing animals, of the water soluble material from smoke condensate is not significantly different (95% confidence limit) from untreated or solvent controls in our experiments, a result which is not surprising in view of the fact that few water soluble mouse skin carcinogens are known. However, that even this low activity cannot be detected in the ethereal extract (Fraction E) would appear to exclude the possibility of simple carcinogenic unsaturated $\gamma$ or $\delta$ lactones of the type found in some plants (Dickens and Jones, 1961) being present in tobacco and passing unchanged into cigarette smoke. The numbers of tumour bearing animals produced by the recombined water soluble (B) and water insoluble (C) fractions were just significantly greater than those from Fraction C alone after 48 weeks and 64 weeks but the differences reduced to insignificance towards the end of the experiment. The number of carcinoma bearing animals was significantly greater for the recombined fractions from 64 weeks onwards. These results suggest that some of the water soluble constituents may act as promoting agents. This interpretation would agree with the finding of earlier workers (Roe *et al.*, 1959; Wynder and Hoffmann, 1961) that the recoverable fractions other than neutral fraction show promoting activity, as some 80% w/w of these latter fractions have been found to be water soluble in our own work. It has been suggested (Demisch and Wright, 1963) that the material responsible for the low tumorigenic activity of the acidic fraction recovered from the alkaline extract of smoke condensate might be PArH solubilised by a mechanism comparable with the solubilisation of hydrocarbons by alkaline solutions of desoxycholic acid. A similar mechanism might afford an alternative explanation for the very low tumorigenic activity in Fraction B. The results of Experiments 2, 5 and 8 show that, apart from the relatively insignificant tumorigenic material extracted into Fraction B and the even smaller amount extracted in Fraction F, nearly all the carcinogens of whole smoke condensate pass into Fraction G *via* Fraction C.

Considerable interest has been centred in the PArH present in tobacco smoke condensate, partly because of the known carcinogenic properties of some members of this group of compounds and, because in the work to date, the fractions containing the recovered activity have been those in which PArH have been concentrated (*loc. cit.*). Wynder and Hoffmann (1968) state that " Tumour initiating activity was found essentially only for those sub-fractions which contain four and five-ring aromatic hydrocarbons. The data now available warrant the conclusion that cigarette smoke contains more types of polynuclear aromatic hydrocarbons than have so far been identified. A significant part of the initiating activity of tobacco tar is certainly due to the presence of such hydrocarbons " and they suggest that hydrocarbons of the chrysene type, and derivatives thereof, account for a significant proportion of this activity.

Sub-fractionation of Fraction G involving the stages of urea adduction, distribution between cyclohexane and dimethyl sulphoxide and adsorption with deactivated silica gel (Scheme 1, Fig. 2) yielded a sub-fraction (N) which, it is thought with some certainty from radiochemical evidence, should contain all the three-, four- and five-ring PArH and probably the larger members of the group. The results given in Table V show that Fraction N is the only fraction with significant activity: a result which appears to confirm Wynder and Hoffmann's observations given above. However, this activity is equivalent to no more than 12% of that given by SWSC when both are tested at 300 mg./wk (equiv. dose) and even at double this dose, 600 mg./wk (equiv. dose), Fraction N only produces half the tumour yield of SWSC (at 300 mg./wk (equiv. dose)). Fractions K and M showed only little activity and Fraction H showed a small but significant activity.

These results can be explained in two ways:

1. Considerable amounts of carcinogenic constituents of SWSC are lost in the three stages of separation between Fractions G and N.
2. Initiators and promotors are present in Fraction G and in the subsequent fractionation procedures the promoters are removed into one or all of the Fractions H, K or M whilst the initiators, included amongst which are the PArH, pass through to Fraction N. That the recombined Fractions E + F + H + K + M (the PArH-free fractions except D) gave an increased incidence of both tumour bearing animals and more particularly carcinoma bearing animals over the only fraction (H) to give a significant activity in both respects when tested alone, might be explained by the separation of promoting material in one or more of these fractions other than Fraction H.

These possibilities are being examined in a comprehensive series of mouse skin painting experiments in which all the fractions in Scheme 1 from SWSC through to Fraction N are being tested individually and in recombination. Full details of this work will be published when complete, but results after 64 weeks already strongly indicate that there is no major loss of tumorigenic activity up to and including the separation of Fraction L. Furthermore, those tests which duplicate earlier work (Table V) are giving the same results at the same stage of the experiments. The recombined fraction, M + N, tested at 600 mg./wk (equiv. dose) after 56 weeks, has produced 10·8% animals with tumours and the recombined fraction, H + K + M + N, has produced 11·8% compared with 12·8%, 29·3% and 31·3% for Fractions N, L and G respectively, tested alone at the same dose level. These results, even at this early stage of the experiments show that considerable losses have occurred in the final stage (5) of the separations. It is thought that carcinogens other than PArH are irreversibly adsorbed on, or catalytically destroyed by the silica gel.

A similar loss of activity is shown in the results from tests on the hydrocarbon fraction (HC) and SWSC. In all our experiments at 300 mg./wk (equiv. dose), neutral fraction has produced 70–80% the yield of animals with tumours produced by SWSC at this level. The hydrocarbon fraction produced only 30% of the tumour bearing animal yield of SWSC although the fraction contained all the PArH of smoke. The further concentration of PArH by counter-current extraction into sub-fraction HC1 produced material having the same activity as the PArH Fraction N produced by a totally different method.

The direct application of the counter-current extraction technique to Fraction G (Scheme 2, Fig. 1) results in the concentration of unsubstituted PArH into sub-fraction G1 which has similar activity to that of the two other PArH-rich fractions, N and HC1, the yields of tumour bearing animals being 7·6%, 6·8% and 5·2% for Fractions G1, HC1 and N respectively. In this separation, however, the PArH-free sub-fraction, G2, is considerably more active (28·2% tumour bearing animals) than the PArH-rich fraction, G1, a result which contrasts with those from other schemes. It seems likely that virtually all the biologically active material in sub-fraction G2 is lost in the alternative separation scheme between Fractions L and N and a high proportion of it is lost in the separation of the hydrocarbon fraction (HC) from neutral fraction. This active material appears to be irreversibly adsorbed on silica gel and when adsorbed on alumina it is only possible to elute a proportion of the residue, left after removal of hydrocarbon fraction, with methanol. In one experiment this material when tested by mouse painting gave half the percentage of animals with tumours given by the hydrocarbon fraction. The tumour yield from the recombined Fractions G1 and G2 was 35% as compared with 45·9% for the original Fraction G (Table VI), indicating that the overall loss of active material in the separation is not high.

The low biological activity of the three fractions containing PArH (N, HC1 and G1) has led to the consideration of groups of chemical carcinogens, other than PArH, which might conceivably contribute to the biological activity of tobacco smoke condensate. A few members of two such groups of compounds, closely related to the PArH, namely alkyl substituted aromatic hydrocarbons and heteropolycyclic aromatic compounds, have been detected in smoke condensate (*e.g.* Grob, 1965, 1966, and Van Duuren *et al.*, 1960). As a result of the determination of the distribution coefficients of a wide range of both types of compound between the solvent pairs used (Whitehead and Rothwell, 1969, unpublished data) it has been found that most of these would not have been isolated in the PArH-enriched Fraction HC1 or G1. Methyl derivatives of PArH have distribution coefficients similar to those of the parent compounds but substitution with higher molecular weight alkyl groups raises the values, so that these higher homologues if present in condensate, would be extracted in Fractions G2b and HC2b. Conversely, the majority of the nitrogen-heteropolycylic compounds, having low distribution coefficients would be extracted in Fractions G2a and HC2a. Subfractions of G2a and G2b are at present being tested for mouse skin carcinogenicity to determine, more precisely, the distribution coefficient range in which the non-PArH carcinogenic constituents are to be found.

SUMMARY

1. Three fractionation schemes are described for the concentration of the polycyclic aromatic hydrocarbons (PArH) of cigarette smoke condensate.

2. Schemes 1 and 2 have their first two stages in common which result in the separation of cyclohexane soluble material (Fraction G), representing 24% by weight of the original smoke condensate, from water soluble and methanol soluble constituents.

3. The mouse painting tests show that by direct comparison Fraction G contains a very high proportion of the tumorigenic components of stored non-volatile whole smoke condensate (SWSC). Neutral fraction (33–35% w/w SWSC) at 600 mg./wk (equiv. dose) gives a similar number of both tumour bearing animals and carcinoma bearing animals as Fraction G (24% w/w SWSC) at 480 mg./wk (equiv. dose). Fraction G is considered to be the more satisfactory material for further attempts to isolate the mouse skin carcinogenic factors in whole smoke condensate.

4. Fractions N, G1 and HC1, the materials containing the PArH in most concentrated forms so far tested for biological activity, produced similar numbers of animals with tumours in each case.

5. In fractionation Scheme 1 considerable losses of carcinogenic material, which are not reflected in losses of PArH, occur during the last three stages of the separation. Recent work indicates that this loss occurs by irreversible adsorption or catalytic destruction of biologically active material on silica gel. Similar, but less pronounced, losses were encountered in the preparation of hydrocarbon fraction from neutral fraction (Scheme 3) by the partially irreversible adsorption or destruction of biologically active material on alumina.

6. Scheme 2 gave some loss of biologically active material but this was not appreciable. Whereas Fraction G1, containing all the PArH, proved to be as active as Fractions N and HC1, its activity was only one-quarter of that of Fraction G2, the recovered PArH-free material and one-sixth of the activity of whole smoke condensate.

7. There was no effect on the incidence of spontaneously occurring tumours or disease processes in any of the treated groups as compared with untreated control mice housed under identical conditions.

8. The general conclusions drawn from this work are that solvent partition methods for the fractionation of SWSC are highly satisfactory in minimising losses of biologically active material and that the unsubstituted polycylic aromatic hydrocarbons are probably not such important carcinogenic constituents of cigarette smoke condensate as has been thought hitherto.

The authors thank the Tobacco Research Council for permission to publish this paper, Doctors T. D. Day and R. F. Davies for supervising all the biological experiments and post-mortem examinations and for carrying out the histological diagnoses, Mr. P. N. Lee for statistical assistance, Mr. T. Smith for supervising the large scale preparation of fractions and Mr. D. V. D. Thorowgood who was responsible for the animal husbandry.

### REFERENCES

Bonnet, J. and Neukomm, S.—(1957) *Helv. chim. Acta*, **40**, 113.
Davies, R. F. and Day, T. D.—(1969) *Br. J. Cancer*, **23**, 363.
Day, T. D.—(1967) *Br. J. Cancer*, **21**, 56.
Demisch, R. R. and Wright, G. F.—(1963) *Can. J. Biochem. Physiol.*, **41**, 1655.
Dickens, F. and Jones, H. E. H.—(1961) *Br. J. Cancer*, **15**, 85.
Elmenhorst, H. and Grimmer, G.—(1968) *Z. Krebsforsch.*, **71**, 66.
Gellhorn, A.—(1958) *Cancer Res.*, **18**, 410.
Grimmer, G.—(1962) *Beitr. Tabakforsch.*, **1**, 291.
Grob, K.—(1965) *J. Gas Chromatogr.*, **3**, 52.—(1966) *Beitr. Tabakforsch.*, **3**, 403.
Gwynn, R. H. and Salaman, M. H.—(1956) *Rep. Br. Emp. Cancer Campgn*, **34**, 193.
Haenni, E. O., Howard, J. W. and Joe, F. L.—(1962) *J. Ass. off. agric. Chem.*, **45**, 67.
Hamer, D. and Woodhouse, D. L.—(1956) *Br. J. Cancer*, **10**, 49.
Hoffmann, D. and Wynder, E. L.—(1966) *Proc. Am. Ass. Cancer Res.*, **7**, 32.
Mold, J. D., Walker, T. B. and Veasey, L. G.—(1963) *Analyt. Chem.*, **35**, 2071.
Neukomm, S.—(1957) *Oncologia, Basel*, **10**, 137.
Roe, F. J. C., Salaman, M. H. and Cohen, J.—(1959) *Br. J. Cancer*, **13**, 623.
Seelkopf, C., Ricken, W. and Dhom, G.—(1963) *Z. Krebforsch.*, **65**, 241.
Tobacco Research Council—(1967) 'Review of Activities, 1963–1966'. London (T.R.C.), p. 40.
Van Duuren, B. L., Bilbao, J. A. and Joseph, C. A.—(1960) *J. natn. Cancer Inst.*, **25**, 53.
Wynder, E. L. and Hoffmann, D.—(1959) *Cancer, N.Y.*, **12**, 1079.—(1961) *Cancer, N.Y.*, **14**, 1306.—(1967) 'Tobacco and Tobacco Smoke'. New York and London (Academic Press), p. 231.—(1968) *Science, N.Y.*, **162**, 862.
Wynder, E. L. and Wright, G. F.—(1957) *Cancer, N.Y.*, **10**, 255.